# Polyelectrolyte Microcapsules: An Efficient and Rapid Adsorbent for Uranine

**DOI:** 10.3390/gels12080743

**Published:** 2026-08-19

**Authors:** Aleksandr L. Kim, Egor V. Musin, Sergey A. Tikhonenko

**Affiliations:** 1 Institute of Theoretical and Experimental Biophysics Russian Academy of Science, Institutskaya St., 3, 142290 Puschino, Moscow Region, Russia; tikhonenkosa@gmail.com; 2 Moscow Polytechnic University (Moscow Polytech), Bolshaya Semyonovskaya Str., 38, 107023 Moscow, Russia; eglork@gmail.com

**Keywords:** layer-by-layer assembly, adsorption kinetics, green wastewater treatment, emerging fluorescent contaminants, sustainable sorbents, PSS, PAH

## Abstract

Fluorescein-based tracers like uranine are extensively used in hydrogeology, textile marking, and cosmetics, yet their discharge generates dilute wastewater streams that challenge conventional treatment due to low removal efficiency and high operational costs. This study evaluates the sorption potential of polyelectrolyte microcapsules (PMCs) fabricated via a green, aqueous layer-by-layer (LbL) assembly on sacrificial CaCO_3_ templates for uranine decontamination. The PMCs achieve rapid equilibrium within ≤5 min, with kinetics governed by the pseudo-second-order model and equilibrium data described by the Langmuir isotherm, yielding a maximum capacity of 12.1 mg/g. Sorption is highly efficient at neutral to mildly acidic pH (3.0–7.0) and low ionic strength (≤0.15 M NaCl), providing 98–100% removal from dilute streams (C_0_ ≤ 10 mg/L) and consistently reducing effluent concentrations to <0.05 mg/L. Saturated capsules exhibit low spontaneous dye release (≤10%) in deionized water under the tested conditions. Although limited regenerability precludes multi-cycle industrial use, the rapid sorption kinetics and high uranine removal efficiency make PMCs well suited for single-use polishing applications. By offering a scalable, solvent-free synthesis and targeted removal of emerging fluorescent pollutants from low-concentration effluents, this work presents a sustainable, low-energy alternative for advanced wastewater treatment, aligning with green chemical engineering and circular water management principles.

## 1. Introduction

Uranine (the sodium salt of fluorescein, C_20_H_10_Na_2_O_5_, MW 376.27 g mol^−1^) is a synthetic anionic xanthene dye characterized by high water solubility (≥150 g L^−1^ at 25 °C), a low log Kow (≈−1.2), and a fluorescence quantum yield of 0.85–0.93 [1]. In hydrogeology, it is employed as a hydrodynamic tracer (detection limit 10^−9^–10^−10^ g L^−1^, typical dosage 0.1–5.0 g km^−3^) for mapping subsurface flows and localizing leaks [2,3,4]. Over 600 tracer tests are conducted annually across the EU and North America, with residual releases amounting to 15–40 t yr^−1^ [5]. Industrially, uranine serves as a luminescent marker in textiles (constituting 8–12% of fluorescent additives), protective coatings, and cosmetics [6]. In clinical practice, fluorescein sodium (uranine) is a standard agent for retinal angiography (typically administered at 5–10 mg/kg i.v.) and lymphatic mapping in procedures such as sentinel lymph node biopsy for melanoma [7]. Global fluorescein production reaches 10,000–14,000 t yr^−1^; 5–15% enters municipal and industrial effluents, generating streams with concentrations of 2–200 mg L^−1^ that require post-treatment to comply with regulatory discharge limits of <0.05 mg L^−1^ [8,9,10].

Toxicologically, uranine exhibits low acute toxicity (LD_50_ > 4000 mg kg^−1^ in rats); however, chronic exposure to aqueous concentrations >0.5 mg L^−1^ correlates with elevated β_2_-microglobulin and creatinine levels, indicating nephrotoxic potential [11,12]. Solutions exceeding 50 mg L^−1^ induce mucosal irritation, contact dermatitis, and hypersensitivity reactions [13]. In aquatic ecosystems, spectral absorption in the 450–520 nm range attenuates photosynthetically active radiation (PAR) by 15–40% (at 2–10 mg L^−1^), thereby inhibiting phytoplankton photosynthesis (IC_50_: Chlorella vulgaris—3.2 mg L^−1^, Scenedesmus obliquus—4.1 mg L^−1^) and inducing oxidative stress in aquatic organisms (LC_50_ for Daphnia magna—12.4 mg L^−1^, 48 h exposure) [14]. A consequent 20–35% decline in primary productivity disrupts trophic networks and accelerates eutrophication processes [9,14]. Consequently, the development of advanced extraction protocols for uranine to achieve residual concentrations < 0.01 mg L^−1^ represents a pressing objective in environmental engineering.

Contemporary wastewater treatment strategies for synthetic dyes encompass coagulation–flocculation, membrane separation, advanced oxidation processes (AOPs), and adsorption. Coagulation using aluminum and iron salts achieves uranine removal efficiencies of 40–65% at dosages of 20–50 mg L^−1^; however, this approach generates 2–4 kg of sludge per m^3^ of treated water, necessitates subsequent dewatering and disposal, and exhibits sharply diminished efficacy at influent concentrations < 10 mg L^−1^ [15]. Membrane-based technologies (nanofiltration, reverse osmosis) demonstrate rejection coefficients of 92–98% but are accompanied by a 40–70% permeate flux decline within the initial 24 h of operation due to fouling, require transmembrane pressures of 10–20 bar, and yield retentate concentrates containing 5–15 g L^−1^ of dye, the management of which is economically unviable [16,17]. AOPs (ozonation, UV/H_2_O_2_, Fenton chemistry) achieve >90% degradation at specific energy consumptions of 0.8–2.5 kWh m^−3^; however, incomplete mineralization yields recalcitrant intermediates (resorcinol, phthalic acid, 4-hydroxyphthalic acid) exhibiting comparable or elevated toxicity toward standard bioassays [18]. Adsorption remains the most extensively investigated paradigm owing to its operational simplicity, regenerability, and straightforward scalability. Conventional activated carbons (AC) exhibit equilibrium capacities of 80–150 mg g^−1^ at initial concentrations of 50–200 mg L^−1^; however, their procurement cost (~$0.8–1.2 kg^−1^) fails to capture the full economic burden: thermal regeneration at 800–900 °C consumes 1.5–2.5 kWh kg^−1^ of adsorbent, degrades the porous architecture by 15–25% per cycle, and the disposal of spent carbon bearing residual dye is classified as hazard class 3–4 waste [19,20]. Furthermore, ACs possess inherently low selectivity in the presence of natural organic matter (NOM), which suppresses the effective uranine capacity by 30–50% in complex wastewater matrices [21,22].

In recent years, substantial research efforts have concentrated on engineering functionalized sorbents with enhanced capacity and kinetic activity. Metal–organic frameworks (MOFs), such as UiO-66-NH_2_ and MIL-101(Cr), demonstrate sorption capacities of 210–450 mg g^−1^ driven by coordination interactions between dye carboxylate moieties and open metal sites, alongside π–π stacking with aromatic linkers [23]. Nevertheless, MOFs exhibit pronounced hydrolytic instability at pH > 9 and elevated ionic strengths (>0.1 M NaCl), while their solvothermal synthesis mandates organic solvents (DMF, DEF) and temperatures of 120–180 °C, thereby constraining industrial scale-up [24,25,26]. Carbon nanotubes (CNTs) and graphene oxide (GO) within polymeric composites deliver capacities of 180–320 mg g^−1^ but are susceptible to colloidal aggregation in aqueous media, impede downstream filtration, and pose potential ecotoxicological risks associated with nanoparticle leaching [27,28]. Composite hydrogels based on chitosan and polyacrylamide achieve capacities of 120–280 mg g^−1^; however, extensive matrix swelling compromises mechanical integrity, and equilibrium is typically attained over 2–4 h due to restricted analyte diffusion within the cross-linked hydrogel phase [29,30]. Biochars derived from agricultural residues (coconut shells, rice husks, sawdust) are characterized by moderate capacities of 50–120 mg g^−1^ and low feedstock costs yet exhibit high heterogeneity in porous architecture and insufficient batch-to-batch reproducibility [31,32,33,34]. A unifying limitation across these material classes is the difficulty of structural regeneration without performance decay, secondary waste generation, and diminished efficacy in multicomponent matrices representative of real effluent streams [35].

Polymeric sorbents offer distinct advantages in terms of feedstock accessibility, mild synthetic protocols, and the programmability of surface charge and chemical functionality [36]. Prominent among these are polyelectrolyte microcapsules (PMCs), conventionally fabricated via layer-by-layer (LbL) electrostatic assembly of oppositely charged polyelectrolytes (e.g., PSS/PAH, chitosan/alginate) onto sacrificial templates (CaCO_3_, SiO_2_), followed by template dissolution [37,38,39]. Despite widespread PMC deployment in biomedical delivery and chemical sensing [40,41,42], their application in sorptive dye remediation remains underexplored [43]. It is well documented that planar polyelectrolyte films effectively sequester fluorescein via electrostatic and π–π interactions, routinely employing it as a permeability and local pH probe [44]. Theoretically, PMCs minimize intraparticle diffusion resistance and enable precise modulation of volumetric charge density and hydrophilicity [45,46,47,48,49]. Because the layer-by-layer assembled PAH/PSS shell constitutes a highly hydrated, supramolecular polyelectrolyte hydrogel network [37,45,50], it is hypothesized that cationic domains distributed throughout the bulk polyelectrolyte matrix will electrostatically complex anionic uranine (predominantly in its dianionic form at pH > 7), with supplementary stabilization from hydrogen bonding and hydrophobic partitioning [51,52]. However, quantitative datasets concerning equilibrium capacity, sorption kinetics, and the influence of key operational and matrix parameters on the PMC–uranine system are currently absent from the literature, thereby establishing the scientific novelty of the present investigation.

The primary objective of this study is to experimentally characterize the sorption behavior of polyelectrolyte microcapsules toward uranine and to assess their technological viability for the polishing of dye-contaminated aqueous systems. The resulting data will provide a quantitative foundation for evaluating PEMCs against conventional sorbents and delineate the operational boundaries for their implementation in advanced tertiary wastewater treatment processes.

## 2. Results and Discussion

**Figure 1:**
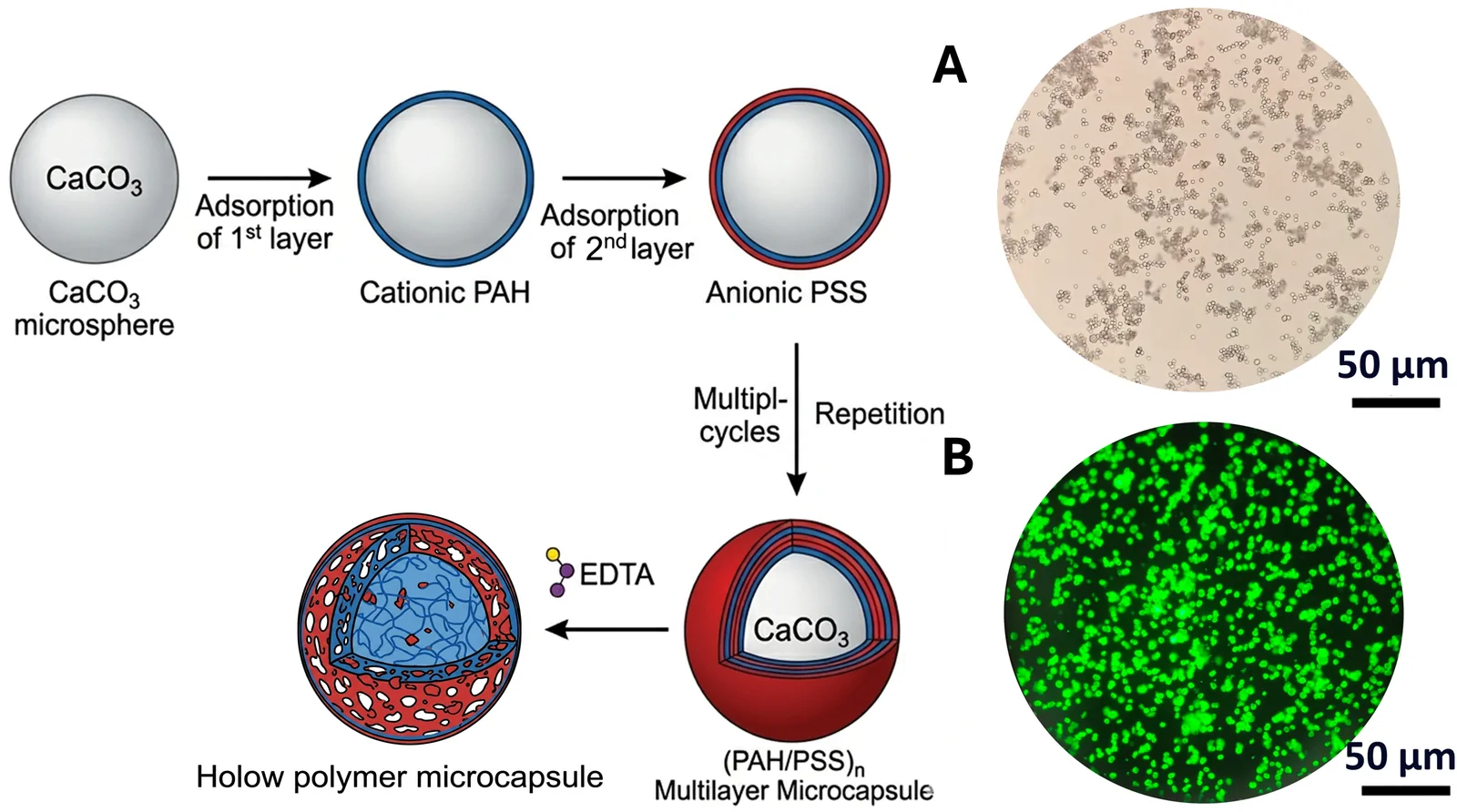
Schematic illustration of polyelectrolyte microcapsule fabrication via layer-by-layer assembly on a carbonate template, accompanied by optical micrographs: (**A**) as-prepared PMCs in aqueous suspension; (**B**) PMCs after reaching sorption equilibrium with uranine (C_0_ = 10 mg L^−1^, 30 min). Synthesis conditions: sequential PAH/PSS deposition from 0.5 M NaCl, template removal in 0.2 M EDTA. Scale bar: 50 µm.

The fabrication of polyelectrolyte microcapsules (PMCs) was carried out via layer-by-layer (LbL) electrostatic self-assembly of oppositely charged poly(allylamine hydrochloride) (PAH) and poly(sodium styrenesulfonate) (PSS) macromolecules onto carbonate sacrificial templates, followed by chelation-mediated template removal. This approach enables the formation of PMCs featuring a highly developed internal polyelectrolyte network. Visual and microscopic assessment of the PMC–uranine system before and after sorption indicates dye uptake and retention of particle stability across the operational range of conditions. A schematic representation of the synthesis protocol and optical micrographs of the resulting particles are presented in Figure 1.

As evident from the presented images, the as-prepared microcapsules exhibit a spherical morphology and uniform size distribution in suspension, confirming the reproducibility of the LbL assembly protocol and the absence of uncontrolled aggregation during template dissolution. Following exposure to uranine solution, the particles display intense yellow–green coloration while fully retaining their geometric integrity, indicating robust immobilization of the dye within the bulk polyelectrolyte matrix. The absence of visible structural degradation or particle agglomeration after sorption underscores the stability of interpolyelectrolyte electrostatic and hydrogen-bonding interactions under conditions approximating practical application. The high colloidal stability of the dye-saturated sorbent minimizes the risk of clogging in flow-through systems and supports the material’s suitability for integration into batch or continuous sorption modules.

**Figure 2:**
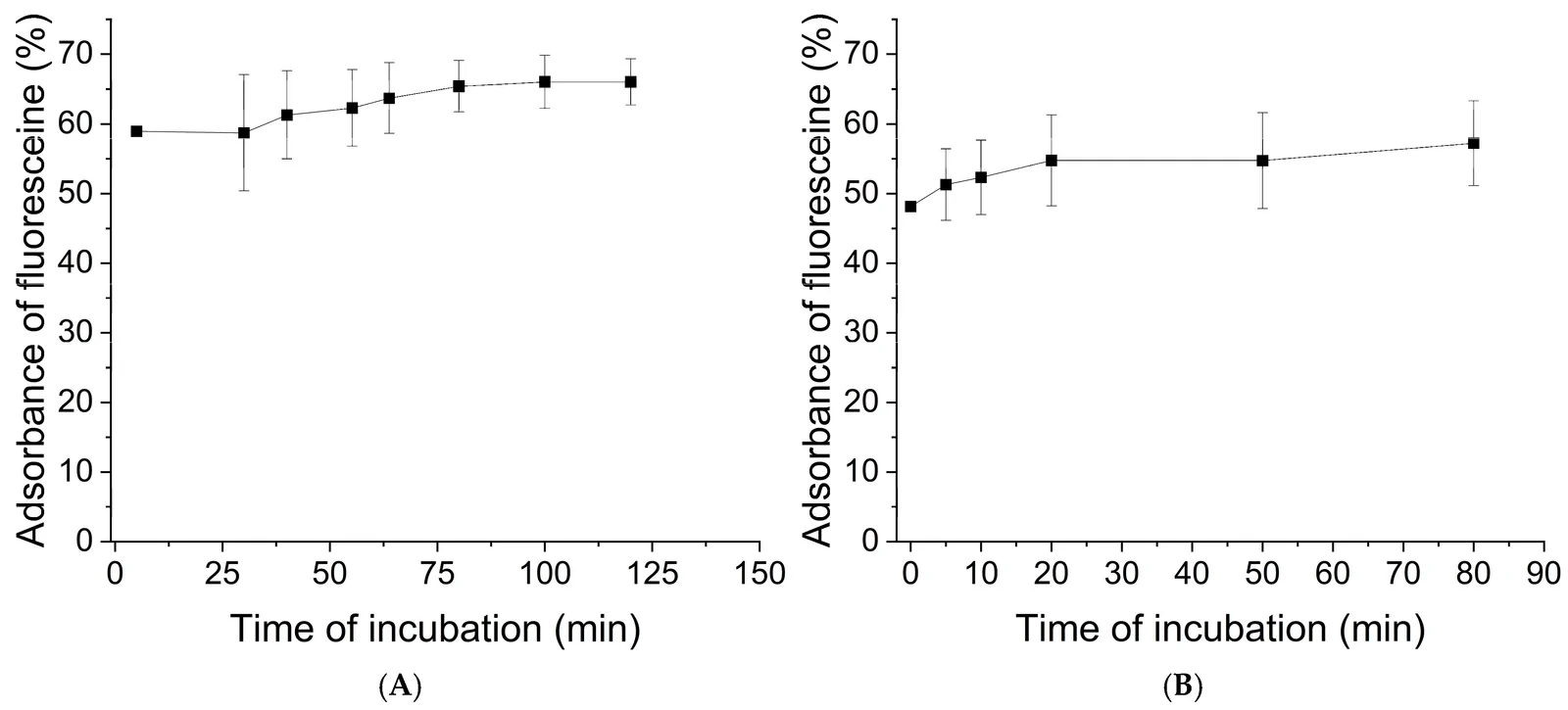
Kinetics of uranine sorption onto polyelectrolyte microcapsules with distinct surface-terminal architectures: (**A**) (PAH/PSS)_2_/PAH, (**B**) (PSS/PAH)_2_/PSS. Conditions: C_0_ = 10 mg L^−1^, T = 25 °C, pH 7.0. Data represent mean ± SD, *n* = 3. The initial amount of uranine before sorption was taken as 100%.

To evaluate the influence of polyelectrolyte layer sequencing on uranine binding dynamics, microcapsules with cationic-terminated polyelectrolyte, (PAH/PSS)_2_/PAH, and anionic-terminated polyelectrolyte, (PSS/PAH)_2_/PSS, were compared. The results are presented in Figure 2.

As shown in Figure 2, the kinetic profiles of dye uptake for both systems exhibit comparable dynamics. Statistical analysis revealed no significant differences (*p* > 0.05) in either the rate of equilibrium attainment or the maximum sorption capacity between the two architectures. This outcome suggests that, under the employed experimental conditions, uranine diffusion into the bulk polyelectrolyte hydrogel framework is not rate-limited by the charge sign of the terminal polyelectrolyte domain but is governed by other physicochemical factors.

**Figure 3:**
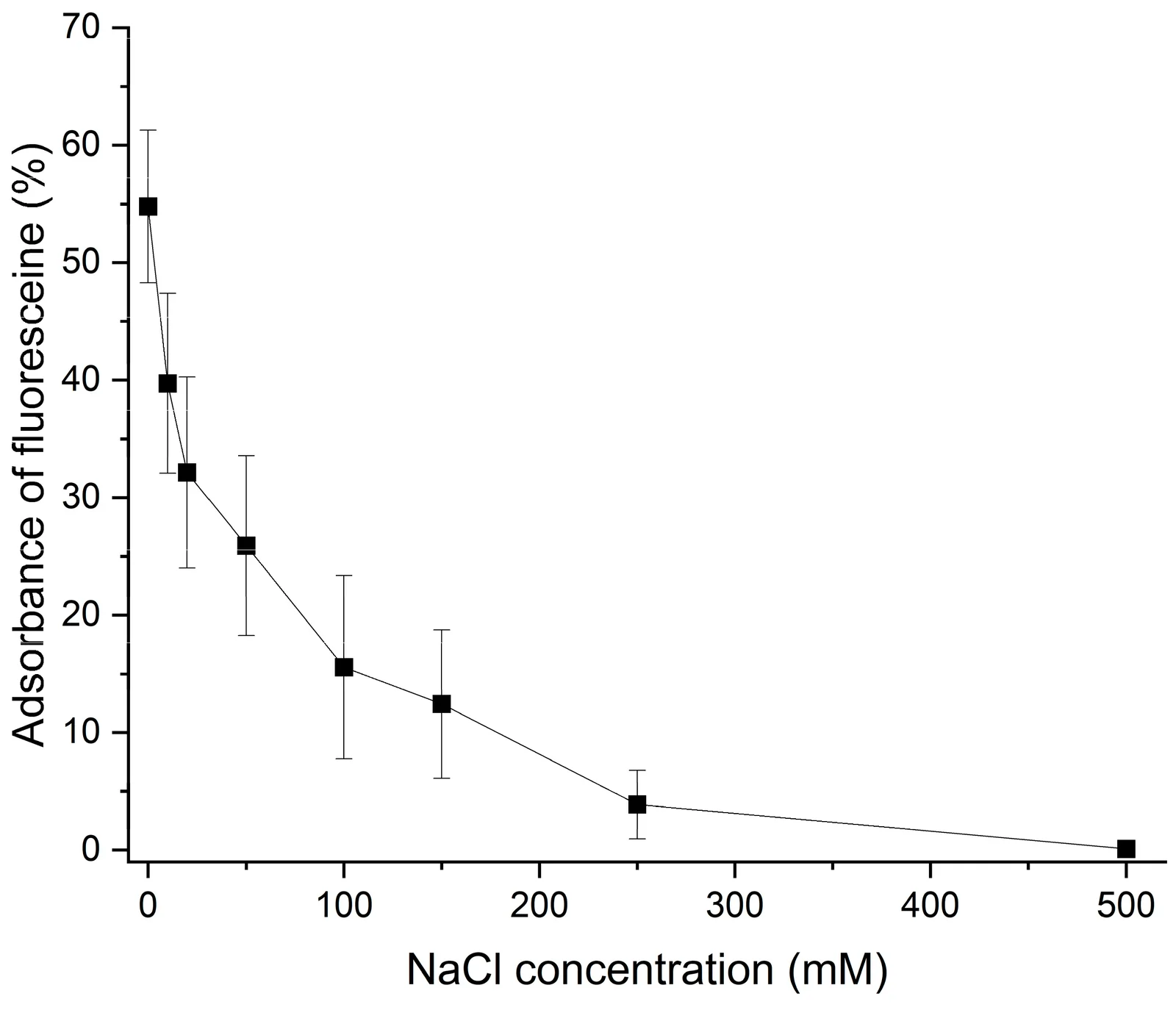
Effect of sodium chloride concentration on uranine adsorption efficiency by polyelectrolyte microcapsules. Conditions: C_0_ = 10 mg L^−1^, T = 25 °C, pH 7.0, contact time = 30 min. Data represent mean ± SD, *n* = 3.

Given that real wastewater and process streams contain dissolved salts, the robustness of the sorption process toward elevated ionic strength was investigated. Experiments were conducted with NaCl concentrations varied from 0 to 500 mM (Figure 3).

A pronounced inverse dependence was established: increasing ionic strength led to a monotonic decline in sorption efficiency. For technological implementation, this defines an upper threshold for the salt content of treatable streams. The application of these microcapsules is most viable at tertiary polishing stages, where salt concentrations do not exceed 0.1–0.15 M, or where preliminary dilution/desalination of effluents is feasible. At ionic strengths > 0.5 M, the removal efficiency falls below operationally acceptable levels, a consideration critical for positioning the sorption module within the treatment train.

The pronounced decrease in adsorption efficiency at ionic strengths exceeding 0.15 M is attributed to the compression of the electrical double layer (Debye screening). The elevated concentration of background electrolyte shields the long-range Coulombic attraction between the anionic uranine species and the cationic domains of the polyelectrolyte matrix, thereby reducing the effective binding affinity.

The observed variability in the sorption efficiency is attributed to the cumulative contribution of several physicochemical factors inherent to batch experiments with colloidal polymeric sorbents. First, the sacrificial CaCO_3_ templates exhibit a natural particle-size polydispersity (4.5 ± 1 µm, CV ≈ 22%), which translates to a distribution of surface-area-to-volume ratios within each PMC batch. Second, the system demonstrates a heightened sensitivity to minor fluctuations in ionic strength in the transition regions, where the electrical double layer compression and protonation states change steeply. Finally, the combined spectrophotometric and dilution uncertainty contributes an estimated ±3–5% to the final concentration values. Importantly, this inherent scatter does not obscure the principal monotonic trends reported herein.

**Figure 4:**
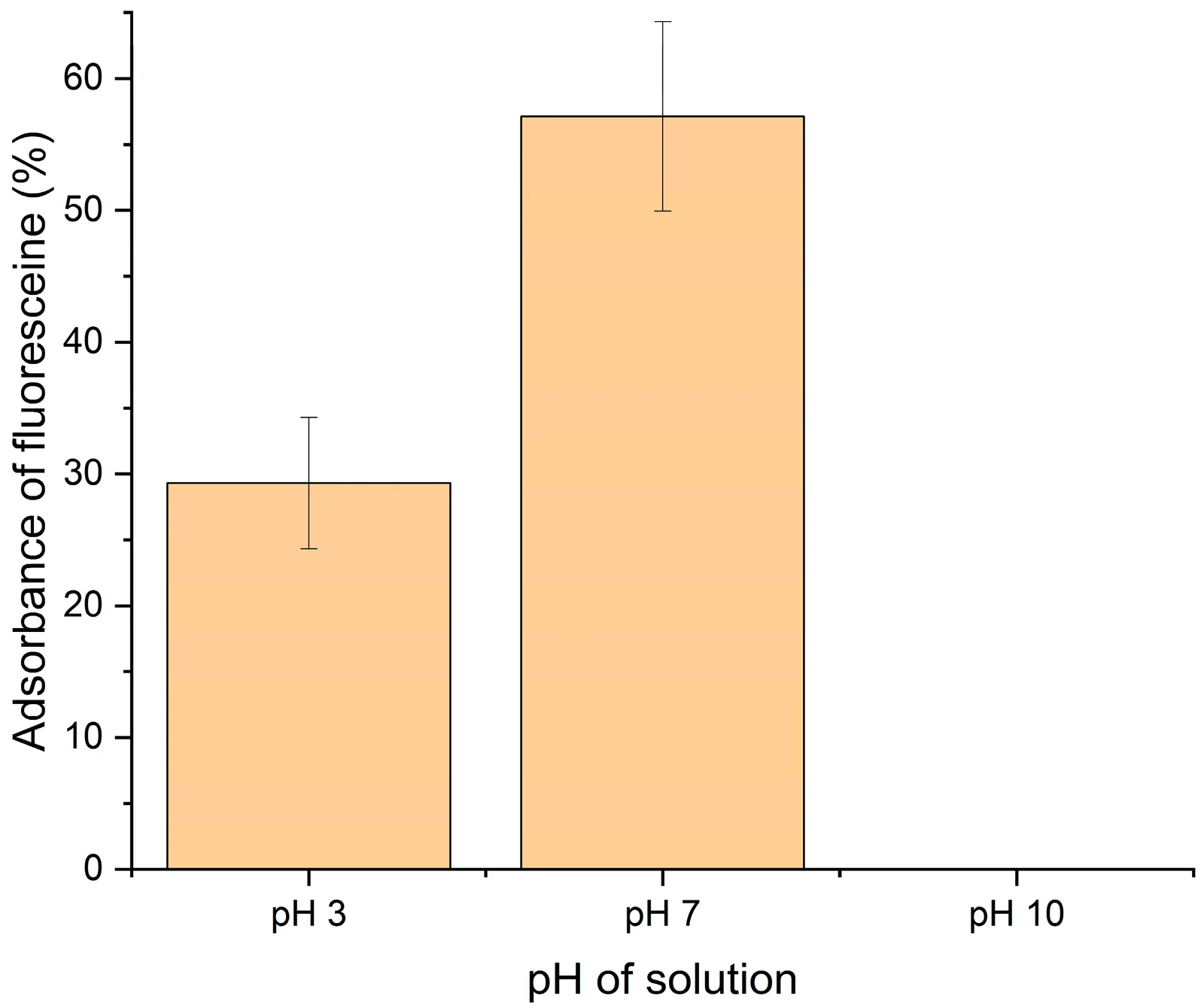
Dependence of microcapsule sorption capacity on aqueous solution pH. Conditions: C_0_ = 10 mg L^−1^, T = 25 °C, contact time = 30 min. Data represent mean ± SD, *n* = 3.

The acid–base regime represents a key operational parameter in optimizing sorption systems. Uranine binding efficiency was evaluated across the pH range 3.0–10.0. Results are presented in Figure 4.

At pH 3.0, adsorption decreases by ~45% relative to neutral conditions, whereas at pH 10.0, sorption is virtually undetectable. These findings delineate the operational pH window of the material as pH 3–7, within which the microcapsules maintain stable performance. Conducting the process under strongly acidic or alkaline conditions would necessitate pH adjustment of the influent, thereby increasing reagent consumption and overall treatment cost. Consequently, the material is optimally suited for processing neutral or mildly acidic process streams without additional chemical conditioning.

**Figure 5:**
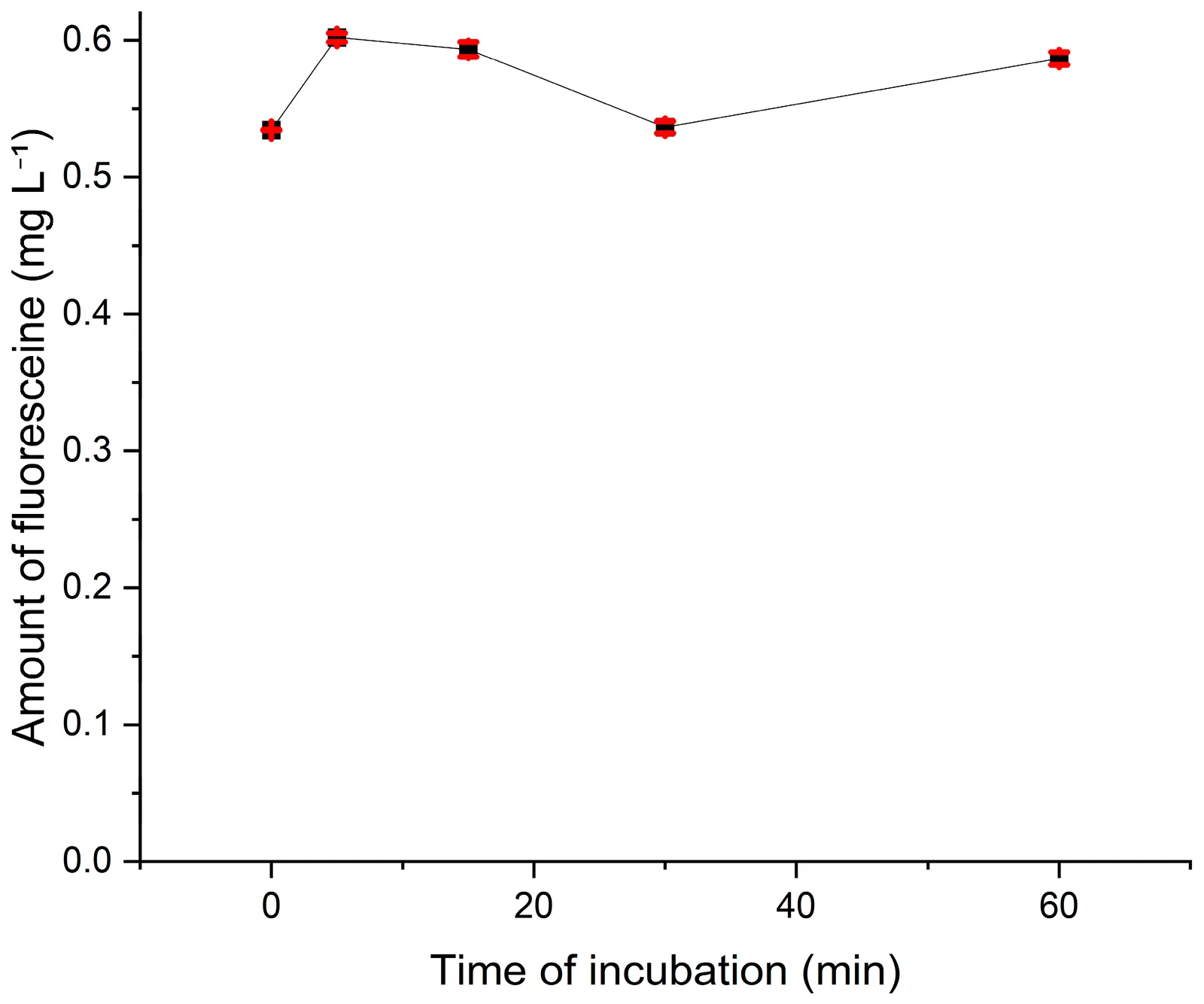
Time course of uranine concentration in the supernatant during incubation of dye-saturated microcapsules in deionized water. Conditions: T = 25 °C. Data represent mean ± SD, *n* = 3.

A critical criterion for sorbent applicability is the absence of secondary contamination due to spontaneous release of the captured species. The stability of the PMC–uranine complex was assessed by transferring dye-saturated microcapsules into deionized water. Results are presented in Figure 5.

The uranine concentration in solution does not exceed 0.6 mg L^−1^ immediately following contact with PMCs and shows no increase during extended incubation, corresponding to ≤10% of the initially adsorbed amount. This level of desorption falls within regulatory limits for discharge of treated water, confirming the environmental safety of employing these microcapsules in open water bodies or recirculating water systems. Dye immobilization within the bulk polyelectrolyte matrix is robust, effectively eliminating the risk of secondary contamination during transport or disposal of the spent sorbent.

**Figure 6:**
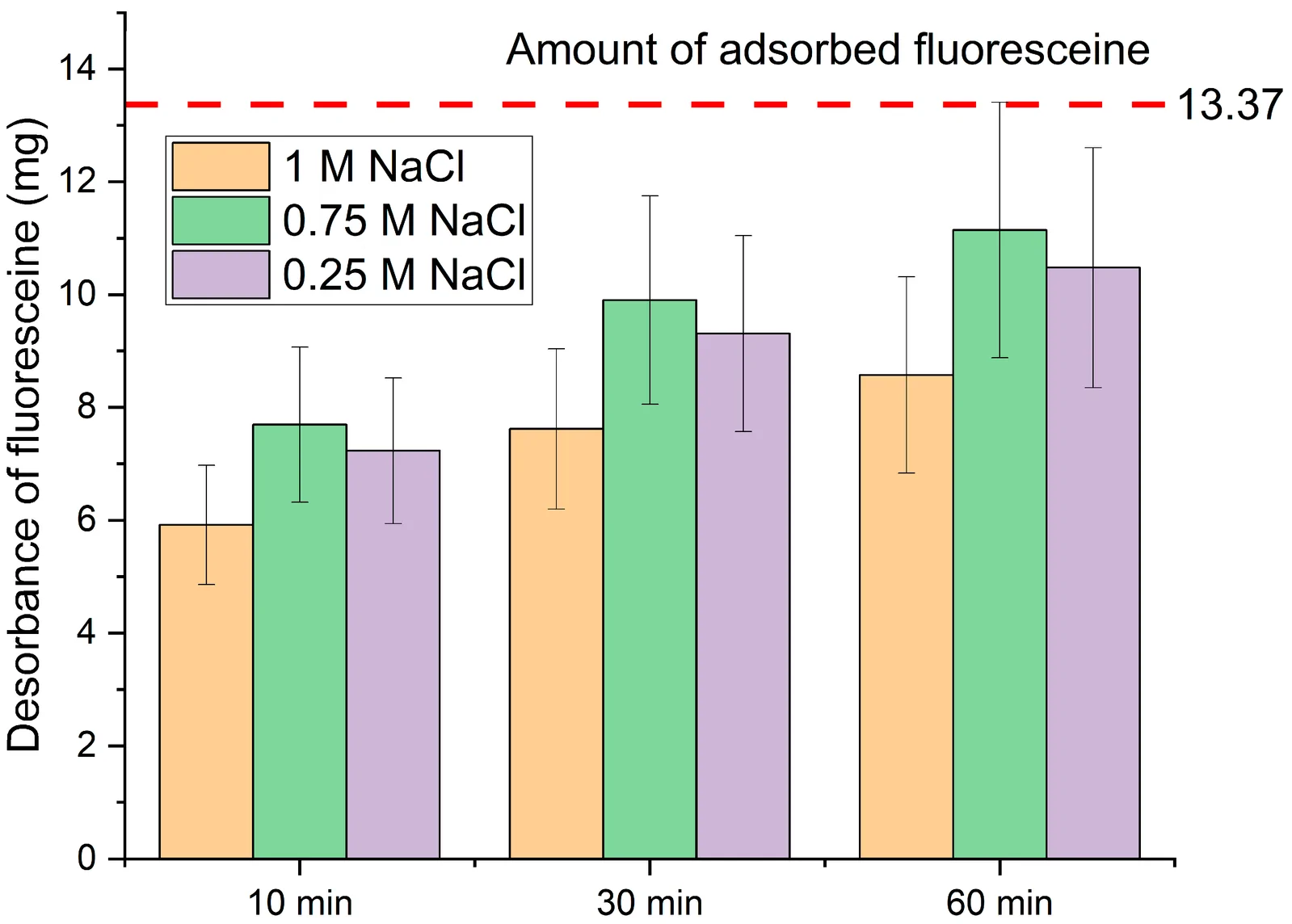
Efficiency of uranine desorption from microcapsules as a function of NaCl solution ionic strength. Conditions: T = 25 °C. Data represent mean ± SD, *n* = 3.

The subsequent experimental phase focused on evaluating the reusability of PMCs and their capacity for repeated uranine sorption cycles. Prior to such assessment, however, conditions for efficient elution of the captured dye from the PMCs must be optimized to maximize regeneration. Increasing ionic strength represents one viable strategy for promoting desorption. Accordingly, NaCl solutions at concentrations of 1.0 M, 0.75 M, and 0.25 M were tested. Results are presented in Figure 6.

Desorption efficiencies of 65%, ~80%, and ~70% were observed at NaCl concentrations of 0.25 M, 0.75 M, and 1.0 M, respectively. The substantial overlap of error bars indicates that the differences between the three concentrations are not statistically significant (*p* > 0.05, n = 3). Re-incubation in fresh eluent of the same concentration did not yield additional recovery. Irrespective of NaCl concentration, a substantial fraction of the sorbed uranine (20–40%) remains irreversibly retained within the bulk polyelectrolyte framework. This incomplete desorption precludes the use of PMCs in closed-loop, multi-cycle operational schemes and confirms their classification as single-use sorbents.

**Figure 7:**
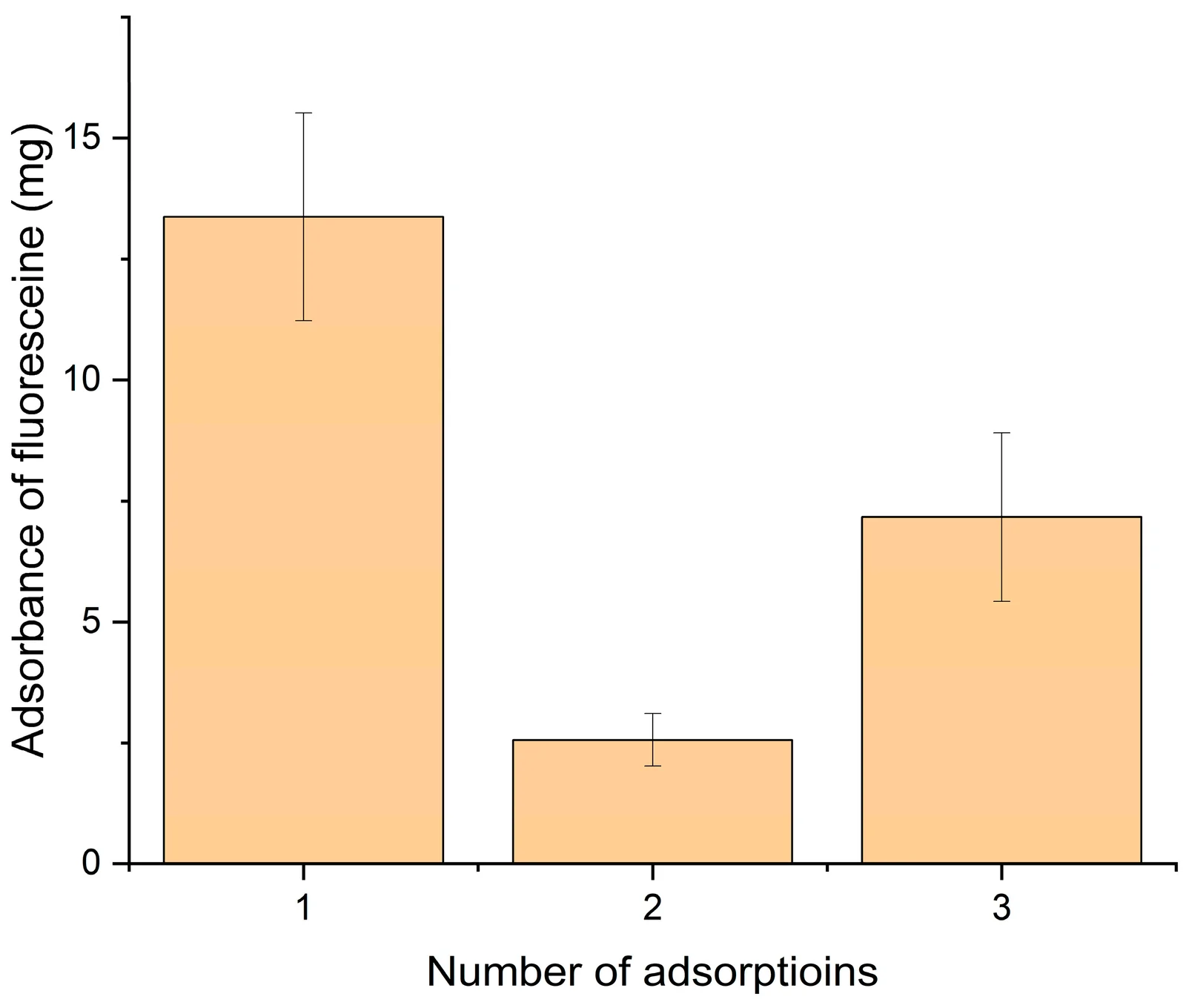
Sorption capacity of polyelectrolyte microcapsules across successive usage cycles. Conditions: C_0_ = 10 mg L^−1^, T = 25 °C, pH = 7.0, desorption in 0.75 M NaCl. Data represent mean ± SD, *n* = 3.

The economic viability of sorption-based technologies is directly contingent upon the material’s ability to retain performance across multiple usage cycles. The sorption capacity of microcapsules following desorption–regeneration cycles was therefore evaluated. Results are presented in Figure 7.

Upon the second usage cycle, the sorption capacity decreases to ≤15% of the initial value. In the third cycle, a partial recovery of activity to ~50% is observed; however, the nonlinear and unstable dynamics render the process unpredictable. Given the significant loss of efficiency after the first cycle and the inability to guarantee capacity recovery, the investigated polyelectrolyte microcapsules are not recommended for application in multi-cycle industrial systems.

**Figure 8:**
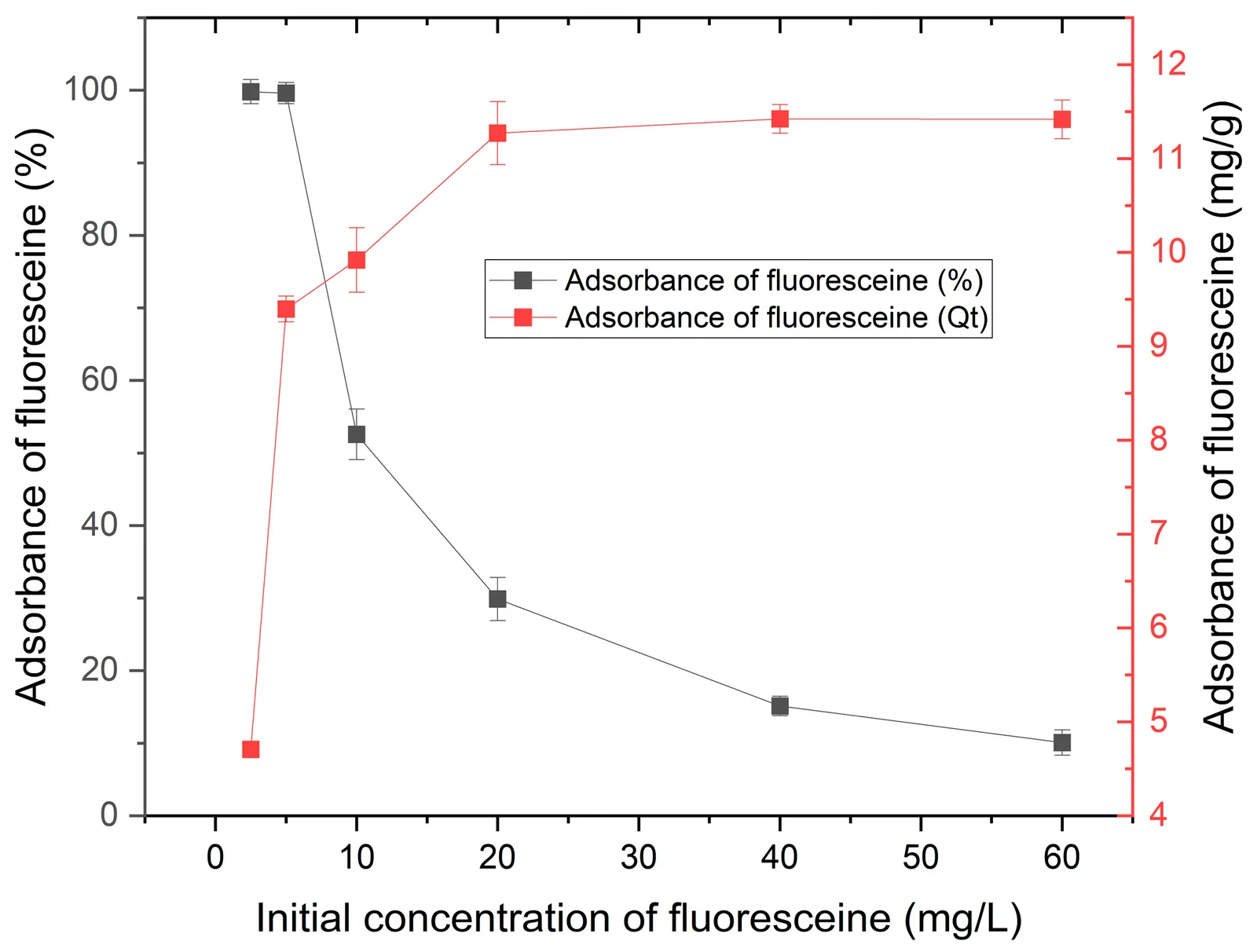
Dependence of uranine adsorption efficiency (%, left axis) and microcapsule sorption capacity (Qt, mg g^−1^, right axis) on the initial dye concentration. Conditions: C_0_ = 2.5–60 mg L^−1^, T = 25 °C, pH = 7.0, contact time = 30 min. Data represent mean ± SD, *n* = 3.

To determine the rational sorbent loading and the effective operational boundaries of the microcapsules, the dependence of uranine adsorption efficiency and sorption capacity on the initial dye concentration in the range of 2.5–60 mg L^−1^ was investigated. Results are presented in Figure 8.

At initial uranine concentrations of 2.5 and 5 mg L^−1^, adsorption efficiency reaches 98–100%. The maximum sorption capacity (Qe = 12.1 mg g^−1^) is recorded at C_0_ = 20 mg L^−1^. Further increases in concentration lead to a decline in adsorption: at 60 mg L^−1^, the value drops to ~10%.

For technological implementation, this defines the rational application range of the microcapsules—treatment of streams with uranine content ≤10 mg L^−1^. The material is most suitably deployed at the final polishing stages of recirculating and discharge waters from textile, printing, and cosmetic manufacturing facilities, where reduction of dye concentration to regulatory levels (<0.05 mg L^−1^) is required.

**Adsorption Kinetic Study**

To identify the rate-limiting step of the sorption process and evaluate the time required to reach equilibrium, a kinetic analysis of the experimental data was performed. Kinetic studies not only elucidate the sorbate–sorbent interaction mechanism but also enable calculation of key parameters essential for process scale-up and the design of sorption units (contact time, throughput, hydraulic loading). In this work, kinetic data were fitted to pseudo-first-order and pseudo-second-order rate equations, which are widely employed to describe the sorption of organic dyes onto polymeric materials [53,54,55].

**Figure 9:**
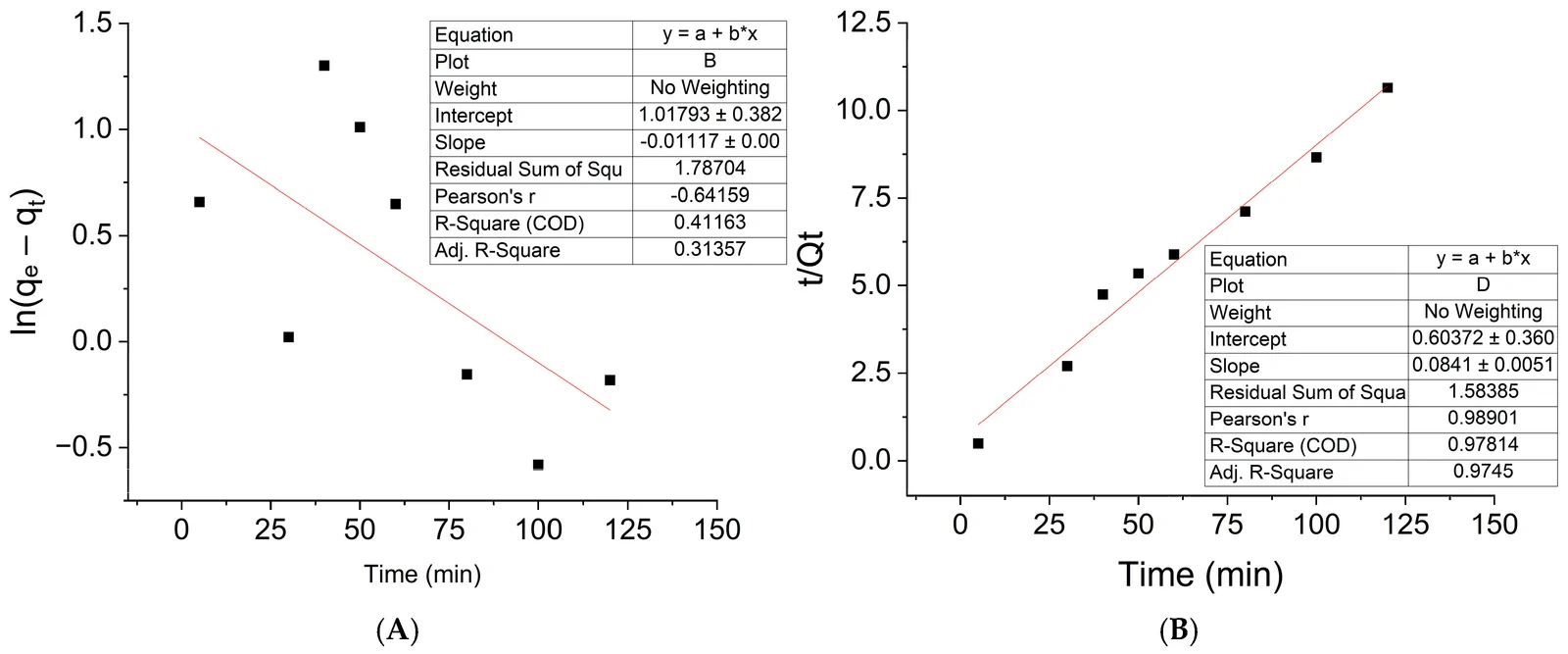
Kinetic modeling of uranine sorption onto polyelectrolyte microcapsules: (**A**) linear regression in pseudo-first-order (Lagergren) coordinates; (**B**) linear regression in pseudo-second-order coordinates. Conditions: C_0_ = 10 mg L^−1^, T = 25 °C, pH 7.0. Data represent mean ± SD, *n* = 3.

As shown in Figure 9A, fitting the experimental data to the pseudo-first-order (Lagergren) equation yields a low coefficient of determination (R^2^ = 0.4116), indicating that this model is inadequate for describing uranine sorption kinetics on the investigated PMCs. This result suggests that the process is not governed by physical adsorption or bulk solution diffusion but is controlled by other mechanistic pathways.

In contrast, the pseudo-second-order model demonstrates excellent agreement with the experimental data (R^2^ = 0.9781; Figure 9B). The calculated equilibrium sorption capacity (q_e_,calc = 11.8 mg g^−1^) aligns closely with the experimentally determined value (q_e_,exp = 12.1 mg g^−1^), providing an additional criterion for model adequacy. The good agreement with the pseudo-second-order model describes the observed sorption kinetics but does not, by itself, establish the molecular nature of the sorbate–sorbent interaction. However, when considered together with the observed pH dependence, the results are consistent with an important contribution of interactions between anionic uranine species and protonated PAH groups. The derived kinetic parameter (k_2_ = 0.047 g mg^−1^ min^−1^) can be utilized to estimate required contact times in sorption columns during technological scale-up of the process.

**Modeling of sorption isotherms and analysis of mass transfer mechanism**

To elucidate the equilibrium partitioning mechanism of uranine between the liquid and solid phases, estimate maximum sorption capacity, and assess the homogeneity of active sites, experimental isotherm data were fitted to the classical Langmuir, Freundlich, and BET models. Additionally, the Weber–Morris intraparticle diffusion model was employed to analyze the contribution of diffusional processes to the overall sorption kinetics. Application of these models enables quantitative justification of the advantages of polyelectrolyte microcapsules (PMCs) over conventional sorbents and delineates the boundaries of their effective application [56,57].

**Figure 10:**
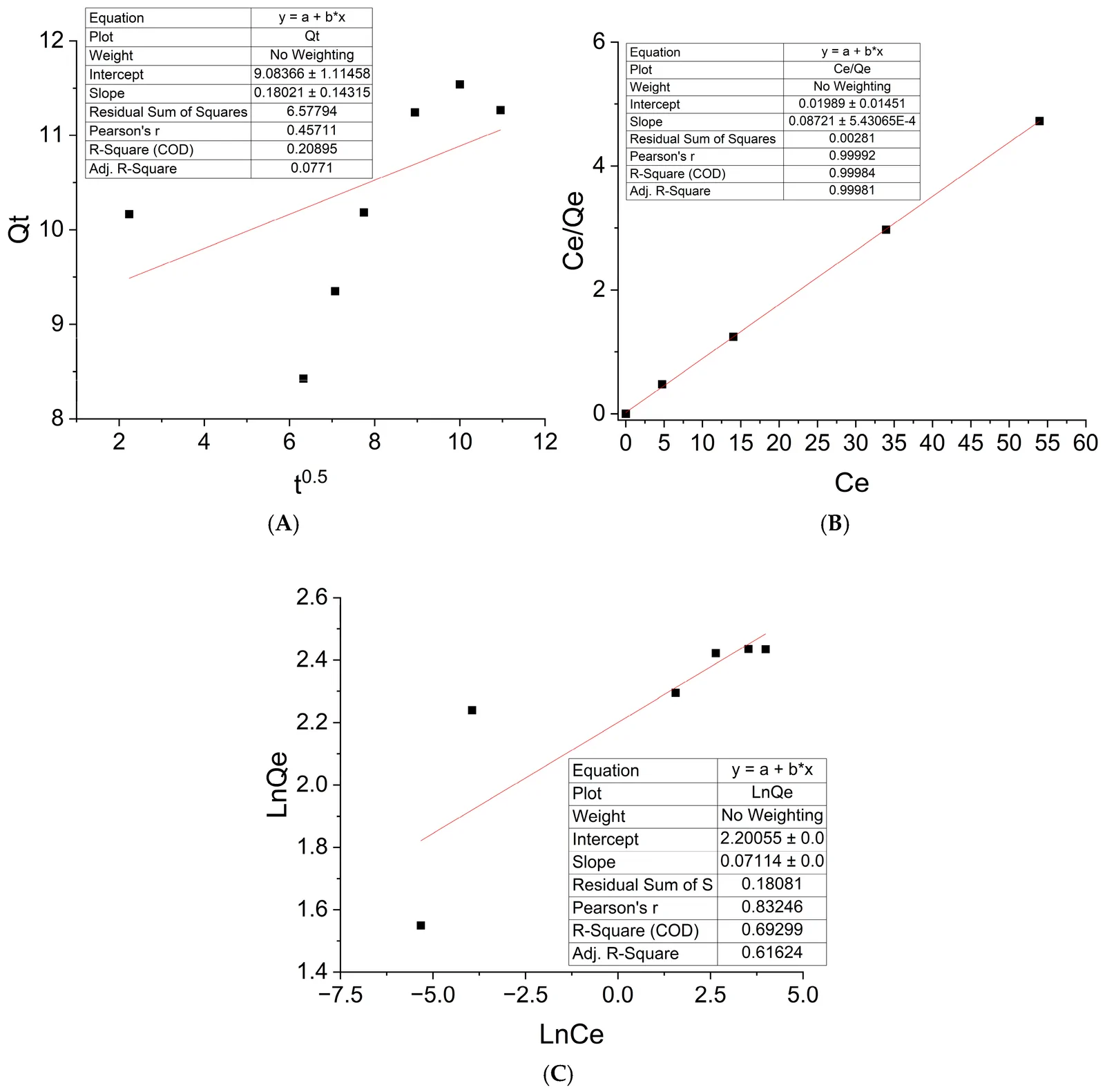
Sorption isotherm modeling and mass transfer analysis of uranine onto polyelectrolyte microcapsules: (**A**) Weber–Morris intraparticle diffusion model; (**B**) Langmuir isotherm; (**C**) Freundlich isotherm. Conditions: C_0_ = 2.5–60 mg L^−1^, T = 25 °C, pH 7.0, contact time = 30 min. Data represent mean ± SD, *n* = 3.

The low R^2^ value (0.2089) indicates that the Weber–Morris (q_t_ vs. t^0^·^5^) linear relationship does not adequately describe the experimental kinetic data under the investigated conditions (Figure 10A). Thus, the data do not support a predominant contribution of the classical intraparticle-diffusion model to the observed kinetics. This result, combined with the well fit of the pseudo-second-order model, suggests that the process is governed by the rate of chemical interaction between uranine and active sites distributed throughout the hydrated polyelectrolyte hydrogel network, rather than by diffusional transport through a discrete barrier. This conclusion aligns with the high permeability characteristic of thin layer-by-layer assembled polyelectrolyte films and the predominance of accessible sorption sites within the near-surface region of the matrix.

The Langmuir isotherm demonstrates exceptional agreement with the experimental data (R^2^ = 0.9998; Figure 10B). The calculated maximum sorption capacity (Q_m_ = 12.1 mg g^−1^) coincides with the experimentally determined value, while the high affinity constant (K_L_ = 5.5 L mg^−1^) is consistent with strong sorbate–sorbent affinity. The excellent fit to the Langmuir model is consistent with preferential sorption on a finite population of energetically similar sites under the investigated conditions. This finding reflects the high structural uniformity of PMCs following LbL assembly, representing a distinct advantage over conventional sorbents possessing heterogeneous porous architectures.

Fitting the data to the Freundlich isotherm yielded a low coefficient of determination when all experimental points were included (R^2^ = 0.693; Figure 10C). The Freundlich parameter n = 2.9 is consistent with favorable sorption; however, the relatively low R^2^ indicates that the Freundlich model provides a substantially poorer description of the experimental data than the Langmuir model.

Collectively, these results suggest that electrostatic attraction between anionic uranine and protonated poly(allylamine) functional groups plays an important role in the sorption process. The observed suppression of sorption at increased ionic strength further supports the contribution of electrostatic interactions. The absence of a rate-limiting diffusional contribution indicates that mass transfer through the hydrated polyelectrolyte matrix does not constitute the dominant kinetic limitation under the investigated conditions. However, these observations provide indirect mechanistic evidence, and direct surface-chemical characterization would be required to conclusively establish the molecular nature of the uranine–PAH interactions.

To quantitatively assess the technological potential of the developed polyelectrolyte microcapsules, a systematic analysis of the kinetic and equilibrium characteristics of uranine sorption was conducted. Based on fitting experimental data to classical models, alongside testing the influence of matrix factors (pH, ionic strength, initial concentration), a parameter set was identified that defines the operational boundaries of the material and the conditions for its integration into water treatment facilities. Summary characteristics required for hydraulic and mass-transfer calculations of sorption modules, as well as for justifying the operational mode (single-use vs. regenerable), are presented in Table 1.

The presented data indicate that uranine sorption onto PMCs is predominantly governed by interactions described by the pseudo-second-order kinetic model and proceeds via monolayer coverage of energetically homogeneous active sites (Langmuir isotherm model). The close agreement between calculated and experimentally determined capacity values (11.8 and 12.1 mg g^−1^, respectively) underscores the high reproducibility of the LbL assembly protocol and the negligible rate-limiting contribution of intraparticle diffusion. This kinetic profile enables the design of sorption columns operating at short hydraulic residence times (≤5 min).

From an operational perspective, the material exhibits optimal performance within the pH range 3.0–7.0, at ionic strengths ≤ 0.15 M, and at initial dye concentrations ≤ 10 mg L^−1^. The limited regenerability (~50% desorption efficiency in 0.75 M NaCl and capacity retention ≤ 15% during the second cycle) shifts the technological paradigm toward single-use sorption configurations. In this operational format, PMCs are most suitably deployed for tertiary polishing of recirculating streams, preconcentration of target analytes from dilute effluents, or as disposable cartridges in decentralized treatment systems, where the economic burden of sorbent regeneration and secondary waste management outweighs the cost of periodic media replacement. These empirically derived parameters provide a foundational dataset for process scale-up and the techno-economic assessment of pilot-scale treatment units.

To objectively evaluate the technological potential of the developed PMCs, their sorption and operational characteristics were benchmarked against conventional and emerging materials reported in the recent literature (Table 2). The comparative analysis encompasses key performance indicators: equilibrium capacity, sorption kinetics, resilience to matrix effects, regenerability, and economic viability. This benchmarking delineates the optimal application niche for PMCs within water treatment frameworks targeting fluorescent dye removal.

The comparative analysis presented in Table 2 indicates that polyelectrolyte microcapsules (PMCs) occupy a distinct technological niche among contemporary sorbent materials. A key advantage of PMCs is their exceptionally rapid kinetic activity: sorption equilibrium is attained within ≤5 min, which is 1–3 orders of magnitude faster than conventional activated carbons, biochars, and inorganic sorbents that typically require hours to days. The capsules are synthesized via aqueous layer-by-layer electrostatic assembly without organic solvents, toxic reagents, or high-temperature treatments, ensuring low production cost, environmental safety, and straightforward industrial scalability. Owing to the homogeneous structure of the polyelectrolyte matrix, the equilibrium data are well described by the Langmuir isotherm (R^2^ = 0.9998) and pseudo-second-order kinetics, indicating the absence of rate-limiting intraparticle diffusion and enabling predictable monolayer immobilization of uranine. In dilute effluents (C_0_ ≤ 10 mg L^−1^), PMCs achieve 98–100% dye removal, consistently reducing residual concentrations to <0.05 mg L^−1^ without additional pH adjustment across the operational range of 3.0–7.0.

Nevertheless, the application of PMCs is associated with several limitations that define their optimal use cases. The maximum sorption capacity (Qmax = 12.1 mg g^−1^) is substantially lower than that of high-performance MOF structures (up to 668.6 mg g^−1^) and commercial activated carbons (~222 mg g^−1^), rendering PMCs less economically viable for treating concentrated industrial streams. Sensitivity to ionic strength (marked capacity decline at I > 0.15 M NaCl) necessitates placement of the sorption module at a tertiary polishing stage or preliminary dilution/desalination of the influent. The most significant technological constraint is limited regenerability: desorption in 0.75 M NaCl reaches only ~50%, and capacity in the second cycle drops to ≤15% due to strong electrostatic retention and partial rearrangement of the intracapsular polyelectrolyte network. This precludes multi-cycle industrial deployment but concurrently minimizes the risk of secondary contamination (spontaneous leaching ≤ 10%).

Thus, despite moderate capacity and restricted regenerability, PMCs outperform alternative sorbents in terms of processing speed, green synthesis, and uranine retention under the investigated conditions. Their optimal application lies in single-use polishing cartridges or modules for final treatment of recirculating water streams, where short contact time, absence of energy-intensive regeneration, and minimal environmental footprint are prioritized. In such configurations, the economic feasibility of periodic sorbent replacement outweighs the costs of complex chemical or thermal regeneration, positioning PMCs as a sustainable, targeted solution for removing emerging fluorescent contaminants from low-concentration aqueous matrices.

## 3. Conclusions

This study demonstrates that polyelectrolyte microcapsules (PMCs) synthesized via a green, aqueous layer-by-layer protocol represent a highly efficient and kinetically rapid sorbent for uranine removal from dilute aqueous streams. The adsorption process reaches equilibrium within ≤5 min and is well described by pseudo-second-order kinetics (R^2^ = 0.9781), with equilibrium data well described by the Langmuir isotherm (R^2^ = 0.9998). Under optimal operational conditions (pH 3.0–7.0, ionic strength ≤ 0.15 M NaCl), the PMCs achieve a maximum capacity of 12.1 mg/g and consistently remove 98–100% of the dye from dilute matrices (C_0_ ≤ 10 mg/L), reliably reducing effluent concentrations below the 0.05 mg/L regulatory threshold. The observed pH and ionic-strength dependencies, together with the absence of a pronounced intraparticle diffusion limitation, support an important contribution of electrostatic interactions to uranine sorption within the polyelectrolyte hydrogel network. However, direct spectroscopic or surface-chemical characterization would be required to conclusively establish the molecular-level sorption mechanism.

Although limited regenerability (~50% desorption in 0.75 M NaCl and ≤15% capacity retention in subsequent cycles) precludes multi-cycle industrial operation, the rapid sorption kinetics and high uranine removal efficiency make PMCs well suited for single-use polishing applications as a final treatment step for uranine-contaminated aqueous streams. By combining solvent-free synthesis, exceptional kinetic performance, and reliable contaminant sequestration, this work establishes PMCs as a sustainable, low-energy alternative for managing emerging fluorescent pollutants. Future research will focus on engineering salt-resilient polyelectrolyte architectures and validating PMC efficacy in complex, real-world multi-component effluents to accelerate pilot-scale deployment and integration into circular water management frameworks.

## 4. Materials and Methods

### 4.1. Materials and Reagents

Polyelectrolytes: sodium poly(styrenesulfonate) (PSS) and poly(allylamine hydrochloride) (PAH) with a molecular weight of 70 kDa were purchased from Merck (Merck KGaA, St. Louis, MO, USA). The residual monomer content was less than 10%. The purity of each chemical compound was at least 99.9%. Poly(allylamine hydrochloride) (PAH, MW ≈ 70 kDa) and poly(sodium styrenesulfonate) (PSS, MW ≈ 70 kDa) were purchased from Sigma-Aldrich (Merck KGaA, Darmstadt, Germany). Uranine (sodium salt of fluorescein, C_20_H_10_Na_2_O_5_, MW 376.27 g mol^−1^, analytical grade), calcium chloride (CaCl_2_·2H_2_O), sodium carbonate (Na_2_CO_3_), sodium chloride (NaCl), ethylenediaminetetraacetic acid (EDTA), hydrochloric acid (HCl), and sodium hydroxide (NaOH) were obtained from Reakhim LLC (Reakhim LLC, Saint Petersburg, Russia). All solutions were prepared using deionized water.

### 4.2. Synthesis of CaCO_3_ Microspherulites

CaCO_3_ microspherulites were synthesized via a coprecipitation method following an adapted protocol [63]. Equimolar (0.33 M) solutions of CaCl_2_ and Na_2_CO_3_ were rapidly mixed under continuous mechanical agitation, which was maintained for 30 s. The resulting suspension was incubated at ambient temperature without agitation to allow completion of precipitation and structural maturation; the process was monitored by optical microscopy. Following sedimentation, the supernatant was removed by decantation in accordance with established isolation protocols [64], and the solid phase was sequentially washed with deionized water prior to its use as a sacrificial template for the fabrication of polyelectrolyte microcapsules (PMCs). Particle size analysis indicated a mean diameter of 4.5 ± 1 µm, consistent with data from previous syntheses.

### 4.3. Fabrication of CaCO_3_-Templated Polyelectrolyte Microcapsules

PMCs were fabricated via layer-by-layer (LbL) electrostatic self-assembly on CaCO_3_ microspherulites according to established protocols [63]. Spherical CaCO_3_ particles (4.5 ± 1 µm) served as sacrificial templates for the sequential adsorption of poly(allylamine hydrochloride) (PAH) and poly(sodium styrenesulfonate) (PSS) from aqueous solutions (2 mg mL^−1^ polymer, 0.5 M NaCl). After each polyelectrolyte deposition step, the templates were subjected to three centrifugation washing cycles (10,000 rpm, 1 min) in 0.5 M NaCl to remove unbound polymer. Following assembly of the desired number of PAH/PSS bilayers, the CaCO_3_ template was dissolved by chelation in 0.2 M ethylenediaminetetraacetic acid (EDTA) for 2 h. This step initiates the formation of an interpolyelectrolyte complex throughout the internal volume of the particle. Final purification involved three rinses with deionized water to remove residual template components. Particle size was monitored by dynamic light scattering (Malvern Zetasizer Nano ZS, London, UK), confirming a mean hydrodynamic diameter of 4.5 ± 1 µm. Two surface-functionalized architectures were employed in this study: cationic-terminated (PAH/PSS)_2_/PAH and anionic-terminated (PSS/PAH)_2_/PSS.

### 4.4. Quantification of Uranine Sorption

To quantify uranine uptake, 1.06 mg of PMCs (corresponding to approximately 1 × 10^8^ particles [65]) were incubated in 2 mL of aqueous uranine solution at a predetermined initial concentration (2.5–60 mg L^−1^) at 25 °C for 30 min (unless otherwise specified in the Results section). In experiments investigating the effect of ionic strength, NaCl concentration was varied from 0 to 500 mM; for pH-dependence studies, values were adjusted using 0.1 M HCl/NaOH over the range 3.0–10.0. Following incubation, PMCs were separated from the solution by centrifugation. A 20 µL aliquot of the supernatant was serially diluted with distilled water to achieve an optical density within the linear range of the spectrophotometer (OD < 2.2). Absorbance was measured at the wavelength corresponding to the uranine absorption maximum (~491 nm). Uranine concentration was calculated from a pre-established calibration curve, accounting for all dilution factors. The sorption capacity of PMCs (Q, mg g^−1^) and removal efficiency (R, %) were calculated using mass balance Equations (1) and (2):

(1) Q = (C_0_ − C_e_)·V/m

(2) R = ((C_0_ − C_e_)/C_0_) × 100%

where C_0_ and C_e_ (mg L^−1^) are the initial and equilibrium uranine concentrations, V (L) is the solution volume, and m (g) is the mass of microcapsules.

### 4.5. Kinetic Analysis of Sorption

Kinetic data were fitted to pseudo-first-order (Lagergren), pseudo-second-order, and Weber–Morris intraparticle diffusion models, described by Equations (3)–(5):

(3) ln(Q_e_ − Q_t_) = ln(Q_e_) − k_1_t

(4) t/Q_t_ = 1/(k_2_·Q_e_^2^) + t/Q_e_

(5) Q_t_ = k_id_·t^0.5^ + C

where Q_e_ and Q_t_ (mg g^−1^) denote the equilibrium and time-dependent sorption capacities; k_1_ (min^−1^) and k_2_ (g mg^−1^ min^−1^) are the rate constants; k_id is the intraparticle diffusion rate constant; and C is a parameter related to the boundary layer thickness.

### 4.6. Adsorption Isotherms

To elucidate the equilibrium sorption mechanism of uranine onto PMCs, experimental data were fitted to the Langmuir (Equation (6)) and Freundlich (Equation (7)) isotherm models:

(6) Q_e_ = (Q_max·K_L_·C_e_)/(1 + K_L_·C_e_)

(7) Q_e_ = K_F_·C_e_^(1/n)

where C_e_ (mg L^−1^) and Q_e_ (mg g^−1^) are the equilibrium concentration and capacity; K_L_ (L mg^−1^) is the Langmuir constant; Q_max (mg g^−1^) is the maximum monolayer capacity; K_F_ ((mg g^−1^)·(L mg^−1^)^1/n^) is the Freundlich constant; and n is the surface heterogeneity parameter.

### 4.7. Statistical Analysis

For each sorption measurement, mean values and relative standard deviations were calculated. The number of replicates (n) was 3. The statistical significance of differences between capsule architectures and experimental groups was assessed using an independent two-sample Student’s *t*-test; differences were considered statistically significant at *p* ≤ 0.05. Fitting of kinetic and isotherm models, calculation of constants, and graphical plotting were performed using OriginPro 2024 software.

## Figures and Tables

**Table 1:** Summary sorption and operational characteristics of polyelectrolyte microcapsules toward uranine.

Parameters	Value	Model/Conditions	Technological Interpretation
Kinetic parameters			
*q_e_*_,exp_	12.1 mg/g	C_0_ = 10C0 =10 mg/L, t = 5 min, pH 7.0	Baseline sorbent productivity under operational conditions
*q_e_*_,calc_ (PSO)	11.8 mg/g	Pseudo-second-order	Supports the contribution of electrostatic interactions
*k*_2_	0.047 g/(mg·min)	Pseudo-second-order	Key parameter for contact time calculation in column design
Isotherm parameters			
*q_max_* (Langmuir)	12.1 mg/g	Langmuir isotherm	Monolayer coverage of homogeneous active sites; negligible rate-limiting diffusion
*K_L_* (Langmuir)	5.5 L/mg	Langmuir isotherm	High phase affinity; stability of the PMC–uranine complex within the bulk polyelectrolyte matrix
*n* (Freundlich)	2.9	Freundlich isotherm	Model indicates favorable sorption, but heterogeneous model provides inferior fit compared to Langmuir
Operational boundaries			
Optimal pH range	3.0–7.0	Static batch conditions	No pH adjustment required for neutral/mildly acidic effluents
Threshold ionic strength (I)	≤0.10–0.15 M NaCl	Salt interference studies	Recommended integration point: post-desalination or after stream dilution
Working C_0_ range	≤10 mg/L	Q_t_ = f(C_0_) dependence	Adsorption efficiency 98–100%; target effluent concentration <0.05 mg L^−1^ achievable
Regeneration characteristics			
Desorption efficiency	~50%	0.75 M NaCl, 25 °C	Enables dye preconcentration; complete elution not achievable
Capacity retention (cycle 2)	≤15%	Single adsorption–desorption cycle	Not recommended for multi-cycle industrial deployment

**Table 2:** Comparative performance of sorbent materials for uranine removal from aqueous media.

Material	*q_max_*, mg/g	Equilibrium Time	Optimal pH	Tolerance to Ionic Strength (I), M	Regeneration	Cost/Scalability	Technological Limitations
PMCs (this work)	12.1	≤5 min	3.0–7.0	≤0.15	~50%, 1–2 cycles	Low (LbL assembly, aqueous media)	Moderate capacity; single-use operational format
Activated carbon from palm seeds [58]	1111.11	90 min; 68.4% removal already after 15 min	Not reported	Not reported	Not reported	Low-cost biomass precursor; scalability untested	Batch study only; no data on ionic strength, regeneration, cycling, or fixed-bed performance
Cu-isonicotinate MOF, Cu(INA)_2_[NO_PRINTED_FORM] [59]	66.67	360 min	9	Not reported	Not reported	Solvent-free synthesis; potentially scalable	Slow adsorption; reusability and salt tolerance not assessed
Biochars (agricultural residues) [60]	0.015–0.20 (palm/olive tree biochars: ~0.12–0.13)	24 h	5.9–10.1 (precipitation at pH 3–5 limits lower bound)	Not reported	Not reported	Very low; simple pyrolysis at 550 °C, 1 h residence, ~35% yield; earth-mound method for traditional charcoal	Low capacity & high variability; poor wood-biochar performance (SSA < 15 m^2^/g); limited data on interferences and dye-pesticide correlation.
Al/Th-MOF [61]	668.6	60–120 min (pore diffusion-limited)	4.0 (protonated surface enhances electrostatic attraction)	High: retains >80% capacity at I = 0.5 M NaCl (π-π stacking, pore filling dominate)	Feasible: >90% recovery after 8 adsorption–desorption cycles (ethanol/acid elution)	High synthesis cost, lab-scale only; scalability uncertain	Complex synthesis; potential metal leaching; requires dedicated regeneration infrastructure
Zn−Al−Hydrotalcite [62]	16.6	~72 h (3 days equilibration with stirring at 25 °C)	7.5–8.0	Not reported	Not reported	Lab-scale synthesis	Surface-only uptake (fixed CO_3_^2−^), low SSA (15.3 m^2^/g), and mandatory pH control

## Data Availability

Data is contained within the article.
